# Identification and genomic characterization of a novel porcine parvovirus (PPV6) in china

**DOI:** 10.1186/s12985-014-0203-2

**Published:** 2014-12-02

**Authors:** Jianqiang Ni, Caixia Qiao, Xue Han, Tao Han, Wenhua Kang, Zhanchao Zi, Zhen Cao, Xinyan Zhai, Xuepeng Cai

**Affiliations:** China Animal Disease Control Center Veterinary Diagnostic Center, Tianguidastreet 17, Beijing, 102600 the People’s Republic of China; Beijing Entry-Exit Inspection and Quarantine Bureau, No.6 Tianshuiyuan Street, Chaoyang District Beijing, 100026 the People’s Republic of China

**Keywords:** Porcine parvovirus, Retrospective study, Phylogenetic analysis, Pairwise comparison

## Abstract

**Background:**

Parvoviruses are classified into two subfamilies based on their host range: the *Parvovirinae,* which infect vertebrates, and the *Densovirinae,* which mainly infect insects and other arthropods. In recent years, a number of novel parvoviruses belonging to the subfamily *Parvovirinae* have been identified from various animal species and humans, including human parvovirus 4 (PARV4), porcine hokovirus, ovine partetravirus, porcine parvovirus 4 (PPV4), and porcine parvovirus 5 (PPV5).

**Methods:**

Using sequence-independent single primer amplification (SISPA), a novel parvovirus within the subfamily *Parvovirinae* that was distinct from any known parvoviruses was identified and five full-length genome sequences were determined and analyzed.

**Results:**

A novel porcine parvovirus, provisionally named PPV6, was initially identified from aborted pig fetuses in China. Retrospective studies revealed the prevalence of PPV6 in aborted pig fetuses and piglets(50% and 75%, respectively) was apparently higher than that in finishing pigs and sows (15.6% and 3.8% respectively). Furthermore, the prevalence of PPV6 in finishing pig was similar in affected and unaffected farms (i.e. 16.7% vs. 13.6%-21.7%). This finding indicates that animal age, perhaps due to increased innate immune resistance, strongly influences the level of PPV6 viremia. Complete genome sequencing and multiple alignments have shown that the nearly full-length genome sequences were approximately 6,100 nucleotides in length and shared 20.5%–42.6% DNA sequence identity with other members of the *Parvovirinae* subfamily. Phylogenetic analysis showed that PPV6 was significantly distinct from other known parvoviruses and was most closely related to PPV4.

**Conclusion:**

Our findings and review of published parvovirus sequences suggested that a novel porcine parvovirus is currently circulating in China and might be classified into the novel genus *Copiparvovirus* within the subfamily *Parvovirinae*. However, the clinical manifestations of PPV6 are still unknown in that the prevalence of PPV6 was similar between healthy pigs and sick pigs in a retrospective epidemiological study. The identification of PPV6 within the subfamily *Parvovirinae* provides further insight into the viral and genetic diversity of parvoviruses.

## Background

The family *Parvoviridae* encompasses small non-enveloped and negative single-stranded DNA viruses, and includes many human and animal pathogens. Porcine parvoviruses (PPV) are important pathogens that cause reproductive failure in swine, resulting in enormous losses in the pig industry worldwide [1]. Epidemiological studies and diagnostic surveys have demonstrated that PPV was the major causative agent responsible for embryonic and fetal death in swine [2]. Parvoviruses are classified into two subfamilies based on their host range: the *Parvovirinae*, which infect vertebrates, and the *Densovirinae*, which mainly infect insects and other arthropods. The subfamily *Parvovirinae* is currently proposed to be divided into eight genera by the International Committee on the Taxonomy of Viruses (ICTV); i.e., *Protoparvovirus, Amdoparvovirus, Aveparvovirus, Bocaparvovirus*, *Dependoparvovirus*, *Erythroparvovirus*, *Copiparvovirus*, and *Tetraparvovirus* [3,4].

A typical parvovirus genome is 4–6.3 kb in length and contains generally two open reading frames (ORFs), encoding for a non-structural protein (NSP) and capsid protein (VP) [3,5]. An additional ORF; i.e., ORF3, encodes an accessory protein and has been identified in some parvoviruses, such as NP1, an NSP only found in bocavirus and PPV4 [6,7]. In recent years, with the use of molecular assays and pathogen discovery tools, a number of novel parvoviruses belonging to the subfamily *Parvovirinae* have been identified in various animal species and humans, including human parvovirus 4 (PARV4), porcine hokovirus (PPV3), ovine partetravirus, PPV4, and PPV5 [6-13]. Among them, PPV3 and ovine partetravirus have been shown to have a close relationship to PARV4, and currently have been proposed to be classified into the genus *Tetraparvovirus* by the ICTV [3,4]. In addition, PPV4 and PPV5 were both identified in clinical samples from swine herds, forming a distinct branch with bovine parvovirus 2 (BPV2) based on phylogenetic analysis, which led to consideration of these viruses being classified in a novel genus; i.e., *Copiparvovirus* [11,12]. Within parvoviruses, six different phylogenetic groups of parvoviruses have been identified from pigs, including classic PPV, PPV2, PPV3, PPV4, PPV5, and porcine bocaviruses (PBoV) [7-12]. The reported overall prevalence of parvoviruses in pig herds has varied from 6.4% to 20% for PPV2, 9.7% to 12.4% for PPV3, 1.5% to 39.7% for PBoV, and 2.6% to 6.6% for PPV5 [14-17]. The prevalence of PPV and PPV4 among the clinical samples in Chinese swine herds was 17.22% and 2.09%, respectively [11,18].

Timely identification of novel pathogens is of great significance in the diagnosis, control and prevention of emerging human and animal infectious diseases. The development of the sequence-independent single primer amplification (SISPA) method in recent years has allowed the rapid identification of new viruses [19]. With the advent of this method, some human and animal viruses, including bovine parvovirus and bungowannah virus have been discovered [19,20]. Using this method, a novel PPV, which is distinct from any known parvovirus, was identified and five almost full-length viral genome sequences were assembled and analyzed in this study.

## Results and discussion

Initially, three mixed tissues samples (i.e., spleen, kidney, and tonsils) collected from aborted pig fetuses in Beijing, China were tested for suspicious agents associated with reproductive failure including pseudorabies virus (PRV), porcine reproductive and respiratory syndrome virus (PRRSV), PPV, porcine circovirus-2 (PCV2), classical swine fever virus (CSFV), swine influenzavirus (SIV), Japanese encephalitis virus (JEV), and *Brucella suis* using RT-PCR or PCR. All samples were found to be negative for the pathogens mentioned above and a nonspecific etiologic agent associated with sow abortion was detected. To explore whether there were any unknown viruses present in the aborted pig fetuses, a SISPA experiment was conducted [21,22]. The sequencing reads were obtained and subjected to BLAST homology searching with sequences deposited in GenBank (BLASTx and tBLASTx programs; http://blast.ncbi.nlm.nih.gov.blast.cgi). The obtained nucleotide sequences included gene fragments of swine, virus, bacteria, and unknown sequences. Of the unknown nucleotide acid sequences, six sequences ranging in size from 298 bp to 650 bp in length exhibited no significant similarity to database sequences. Furthermore, the deduced amino acid sequences of these sequences exhibited either no putative conserved domains (four of six) or contained putative conserved domains of parvoviruses (two of six; BLASTp, E scores < =10^−9^). These results indicated the presence of a possible novel parvovirus. On the basis of its homology to parvoviruses and its swine host, this DNA virus was tentatively named porcine parvovirus type 6 (PPV6) after the two recently identified PPVs; i.e., PPV4 and PPV5 (data not shown) [12,21]. Attempts to stably passage the PPV in PK15 (swine kidney), Vero (African green monkey kidney), and Marc 145 (fetal rhesus monkey kidney) cells were unsuccessful (data not shown).

To investigate the prevalence of PPV6 in clinical samples and the clinical picture associated with PPV6 infection. A retrospective epidemiological study of PPV6 infection was performed by PCR. A total of 171 field samples (160 of sera and 11 of tissues) were collected from apparently healthy pigs and sick pigs with similar reproductive system symptoms from four provinces in China (Beijing, Jiangsu, Tianjin, and Sichuan) during the period of 2010–2013. Among these samples, 48 specimens collected from farms experiencing reproductive disease included 6 aborted fetuses, 4 piglets, 26 sows, and 12 finishing pigs. Taking into consideration that the infection of porcine parvovirus is asymptomatic in growing pigs and multiparous sows, to investigate the distribution of PPV6 in different age group, one half of sow samples and all samples of finishing pigs were collected from healthy pigs. The other 123 specimens collected from healthy farms were all of finishing pigs and were previously identified as negative for PRV, PRRSV, PCV2, PPV, and CSFV. The results showed the prevalence of PPV6 in aborted pig fetuses and piglets(50% and 75%, respectively) was apparently higher than that in finishing pigs and sows (15.6% and 3.8% respectively) no matter what their health status. Furthermore, the prevalence of PPV6 in finishing pig was similar in affected and unaffected farms (i.e. 16.7% vs. 13.6%-21.7%) (Table 1) [23]. This finding indicates that animal age, perhaps due to increased innate immune resistance, strongly influences the level of PPV6 viremia, which is similar to the study result of PRRSV [24].

**Table 1:** **Detection of PPV6 from field samples using PCR**

**Age**	**Health status of sampled farms**	**Location**	**Type of sample**	**No. positive/no. tested samples**	**Prevalence (%)**	**Overall prevalence (%)**
Aborted fetuses	Reproductive failure	Beijing	MTS*	3/6	50	50
piglet	Reproductive failure	Beijing	Serum or MTS	3/4	75	75
Finishing pigs	Reproductive failure	Beijing	Serum	2/12	16.7	15.6
	Healthy	Beijing	Serum	6/42	14.2	
		Jiangsu	Serum	5/36	13.9	
		Tianjin	Serum	3/22	13.6	
		Sichuan	Serum	5/23	21.7	
Sow	Reproductive failure	Beijing	Serum	1/26	3.8	3.8

MTS*, Mixed Tissues Sample.

It is interesting to note that, in samples from aborted pig fetuses collected in Beijing, only PPV6 was detected. Targeted studies are needed to investigate the role of this virus in the sows with reproductive failure [2]. However, the number of clinical samples detected in this study was limited and more extensive epidemiologic studies are needed to elucidate the precise role of PPV6 as a causal agent for reproductive failure. In recent years, a number of novel parvoviruses belonging to the subfamily *Parvovirinae* have been identified, including PARV, PPV4, and PPV5. Even though these new parvoviruses have been extensively studied, the clinical manifestations of these viruses are still unknown. Confirming a causal relationship between a virus and the observed symptoms is an important but difficult issue that normally requires multiple separate studies to be ultimately resolved [19].

Furthermore, PPV6 was detected in samples from all four provinces suggesting that PPV6 has been circulating in a wide area of China. In light of the importance of swine as a potential source of genetic diversity for parvoviruses, identifying possible counterparts of PPV6 in humans and other animals is important in understanding its epidemiology, evolution, and pathogenesis [1].

Based on SISPA-generated fragment sequences, diverging primers were designed to obtain the intervening portions of the genome. The terminal sequences were then acquired using a 5΄ and 3΄ RACE method [8]. Near full-length genome data were generated from 5 positive samples (i.e., PPV6-BJ, BJ2, SC, JS, and TJ) and deposited in GenBank under accession Nos. KF999681-KF999685. Of these sequenced PPV6 isolates, strain BJ was from aborted pig fetuses derived from herds affected with an epizootic reproductive failure in Beijing, whereas the other four strains (i.e., BJ2, SC, JS, and TJ) were from finishing pigs without any clinical signs, independently identified in Beijing, Sichuan, Jiangsu, and Tianjin.

**Figure 1:**
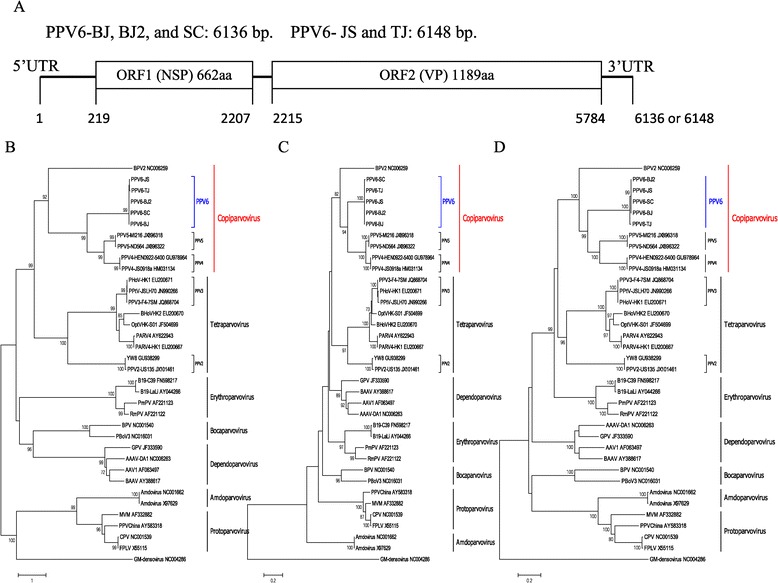
**Genome structure and phylogenetic analysis: bootstrapped neighbor-joining tree based on full-length genomes and NSP or VP amino acid sequences of PPV6 and the** ***Parvoviridae*** **. A**. The solid line represents the DNA genome and the boxes represent the ORFs. The position and size of putative ORFs are indicated. **B**. A phylogenetic tree was constructed based on 5 near full-length genomes (6.1 kb) of PPV6 and 31 genomes from GenBank using amaximum-likelihood method (GTR + G + I) with bootstrap analysis of 1,000 replicates. **C** and **D**. Phylogenetic trees were constructed based on amino acid sequences of NSP and VP using a Poisson correction model with bootstrap analysis of 1,000 replicates. Phylogenetic analyses were conducted using the MEGA6.0 (www.megasoftware.net) program. Branches corresponding to partitions reproduced in <70% bootstrap replicates are collapsed. The evolutionary distance scale is shown at the bottom. The sequence of GM-densovirus (NC004286) is used as outgroup to root the tree. The asterisk indicates the new genus proposed by ICTV.

The near full-length genome of PPV6 contained 6,136 bases (BJ, BJ2, and SC strains) or 6,148 bases (JS and TJ strains) with a G + C content of 46.7–47.1%. Their genome sizes were expected to be larger, and sequencing of the ends was hampered by hair structures. Putative ORFs were obtained using the ORF Finder tool at NCBI (http://www.ncbi.nlm.nih.gov/projects/gorf/) and then were identified by protein blast analysis in the NCBI RefSeq database. The genome organization of PPV6 was similar to that of other parvoviruses, with the characteristic gene order 5′UTR-ORF1-ORF2-3′UTR (Figure 1A) [5]. The ORF1 encodes a putative NSP of 662 amino acids and ORF2 encodes a putative VP of 1,189 amino acids. The predicted sizes of VP (132 kDa) were bigger than most other parvoviruses (most parvoviruses are <90 kDa, including PPV4 and except for BPV2, which was 105 kDa, and PPV5, which was 112 kDa). The sequence similarity between the tested PPV6 strains ranged from 97.1%–99.6% for nucleotides (whole genome) and 97.3%–99.9% for amino acids (NSP and VP) respectively (data not shown). The polymorphic diversity of the VP protein sequences was greater than for NSP protein sequences. The overall amino acid diversity of NSP and VP of five PPV6 strains was 0.0%–0.4% and 0.1%–2.7%, respectively (data not shown). These results suggest that all isolates were closely related to each other genetically.

Five PPV6 sequences were aligned with 31 reference genomic sequences of viruses in the subfamilies of *Parvovirinae* and *Densovirinae* from GenBank using the ClustalX (Ver.1.81) program [25]. Phylogenetic analyses based on the full-length genomes with a maximum-likelihood method (GTR + G + I) showed that PPV6 was distinct from any known parvoviruses and represented a deeply rooted lineage between BPV2 and PPV4 and PPV5 (Figure 1B). The basal phylogenetic position of PPV6 suggested early divergence from other mammalian parvovirus species, and PPV6 may share a common ancestor with PPV4 and PPV5.

Evolutionary trees were also constructed separately for the putative protein sequence of NSP and VP with a Poisson correction model using 500 bootstrap replicates [25]. The topologies of these trees were similar to that of the full-length genome tree (Figures 1C and 1D), and PPV6 formed a distinct cluster within parvoviruses. They also differed from other parvoviruses by their relatively large predicted VP protein. These analyses indicated that PPV6 viruses are likely to belong to the novel genus *Copiparvovirus* within the subfamily *Parvovirinae* [12].

Pairwise comparisons were performed for the nucleotide sequences and predicted amino acids sequences of PPV6 with other parvoviruses. The results showed the genomes of the five strains of PPV6 shared 20.5%–42.6% DNA sequence identity with other members of *Parvovirinae* and are most closely related to PPV4. At the amino acid level, PPV6 exhibited the largest amino acid similarity in NSP with PPV4 (49.8%) and in VP with PPV5 (29.8%; Table 2). PPV6 was found to possess <30% amino acid similarity in the NSP to those of other genera, whereas it exhibited 28.1%–49.8% (BPV2 and PPV4, respectively) identity to that of the genus *Copiparvovirus*. Based on the new criteria for a genus within the subfamily Parvovirinae by ICTV (generally >30% amino acid identical to NS1 proteins within a genus but <30% identical to other genera), the PPV6 would be classified as a novel species of the genus *Copiparvovirus* [4]*.*

**Table 2:** **Nucleotide and amino acid identities of PPV6 with those of other parvoviruses**

**Virus**	**PPV6-BJ**			**PPV4-JS0918a(HM031134)**			**PPV5-ND564(JX896322)**			**BPV2(NC006259)**		
	**Genome(nt)**	**NSP(aa)**	**VP(aa)**	**Genome(nt)**	**NSP(aa)**	**VP(aa)**	**Genome(nt)**	**NSP(aa)**	**VP(aa)**	**Genome(nt)**	**NSP(aa)**	**VP(aa)**
Tetraparvovirus												
PARV4 AY622943	27.5	22.4	17.1	30.5	19.8	17.2	29.5	21.0	19.9	29.3	19.5	18.0
PPV3F4-7SM JQ868704	22.4	14.2	17.3	24.4	11.3	17.5	23.0	12.0	19.8	23.7	12.8	17.3
PPV2US-135 JX101461	27.3	24.1	17.9	30.1	19.3	15.4	27.9	19.7	18.3	27.5	19.0	18.0
Erythroparvovirus												
B19-C39 FN598217	24.8	18.7	14.2	27.7	17.1	16.3	28.5	17.9	17.2	25.6	19.5	14.5
Dependoparvovirus												
AAV1 AF063497	22.5	25.2	11.6	25.7	22.9	15.8	24.6	23.7	14.5	24.5	22.8	13.8
Bocaparvovirus												
PBoV3 NC016031	23.2	15.9	8.9	27.1	15.9	11.7	28.0	16.0	10.8	26.5	16.3	11.3
BPV NC001540	24.1	15.0	9.9	29.5	14.3	11.8	30.5	13.6	11.6	26.7	13.2	11.0
Amdoparvovirus												
Amdovirus X97629	20.5	10.7	6.6	22.8	11.9	7.6	22.0	11.9	9.7	22.5	12.1	8.3
Protoparvovirus												
PPV China AY583318	21.4	15.4	7.9	23.2	15.1	107	22.5	15.0	9.6	23.8	12.8	10.4
Copiparvovirus												
PPV4JS0918aHM031134	42.6	49.8	23.9	100	100	100	62.9	85	38.2	34.6	32.5	19.8
PPV5ND564 JX896322	41.3	49.1	29.8	62.9	85	38.2	100	100	100	33.1	31.9	22.8
BPV2 NC006259	31.7	28.1	18.0	34.6	32.5	19.8	33.1	31.9	22.8	100	100	100
PPV6-BJ	100	100	100	42.6	49.8	23.9	41.3	49.1	29.8	31.7	28.1	18.0
Densovirinae												
GM-densovirusNC004286	24.9	8.5	4.4	24.9	8.4	5.0	24.5	8.8	7.1	25.0	10.2	7.8

All sequence identities were calculated using clustal X. Nt, nucleotide; aa, amino acid.

**Figure 2:**
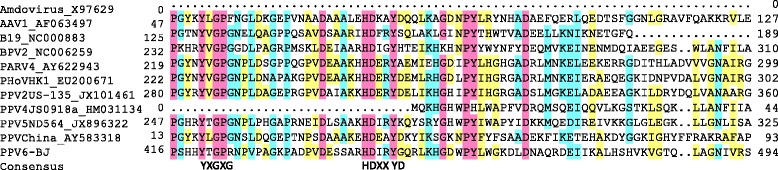
**Sequence alignment of the putative phospholipase A2 motif of PPV6 with those of other parvoviruses.** The conserved amino acids of the Ca^2+^ binding loop (YXGXR) and the catalytic residues (HD--Y) are indicated at the bottom of the alignment. The GenBank numbers of the sequences are indicated.

Even though the overall amino acid homology of PPV6 with other parvoviruses is low, the conserved sequence motifs important for the function of parvoviruses were observed. In the alignment, the conserved replication initiator motif (I and II), NTP-binding and helicase domain (A, B, and C) in the NSP (data not shown) identified within *Parvovirinae* was also found in PPV6 [12,26]. Within the VP unique region, detailed characterization at the amino acid level revealed PPV6 possesses the conserved motifs of the Ca^2+^ binding loop (YXGXR) and the catalytic center (HDXXY) of the putative secretory phospholipase A_2_ (PLA_2_) motif (Figure 2), which are present in the capsid protein of PPV5 but are lacking in PPV4. Furthermore, the conserved motifs of the Ca^2+^ binding loop of PLA2 is the “YXGXR” motif in PPV6, rather than the “YXGXG” or “YXGXF” motif found in most parvoviruses [6]. The PLA2 activity of the alternate motif found in PPV6 needs further study. However, alignment analysis suggested PPV6 is not closely related to any other known human or animal parvovirus, and represents a potential novel species of the genus *Copiparvovirus*, corresponding to the tBLASTx results. Although recombination phenomena have been demonstrated within parvoviral species [27,28], in this study, no recombination signal was found in PPV6 with other parvoviruses by SimPlot analysis (data not shown), which indicated PPV6 is not a recombination of other parvoviruses. Since parvoviruses utilize host DNA polymerase, they are generally considered to be relatively stable, and the increasing identification of these novel parvoviruses with extensive genetic diversity suggests that the evolution of parvoviruses is far more complicated [11,12].

## Conclusion

In summary, we described the identification and genome characterization of a novel parvovirus (PPV6) from pigs, the closest neighbors of which were PPV4 and PPV5. The full genome of PPV6 is approximately 6.1 kb in length, and the genomic organization of PPV6 is similar to PPV5 but not to PPV4, which contains an ORF3 in the middle of the viral genome. However, the genome of PPV6 was slightly distinguished from PPV5 by the larger VP gene and the larger genome size. Phylogenetic analysis demonstrated that PPV6, together with PPV4 and PPV5, form a distinct branch that is genetically different from viruses of previously defined genera in the subfamily *Parvovirinae*, and might be classified into the novel genus *Copiparvovirus* [4,12]. The identification of a novel PPV; i.e., PPV6, within the subfamily *Parvovirinae* provides, further insight into the viral and genetic diversity of parvoviruses. Further study is needed to explore the exact roles of PPV6, especially regarding its host range, geographical distribution, and relatedness to disease [7,9,10,12]. Although PPV4, PPV5, and PPV6 were discovered in recent years, the biological characteristics of these viruses and relatedness to diseases are still not fully understood. Future endeavors to culture PPV6 will help address these questions.

## Materials and methods

### Ethics statement

All samples utilized were originated from sick or healthy pig case submissions to the Veterinary Diagnostic Laboratory of the China Animal Disease Control Center (CADC-VDL) for diagnostic workups. The protocol for this study was approved by the Biosafety Committee of the CADC, Beijing, China.

### Sample collection

All samples were obtained from Beijing, Jiangsu, Tianjin, and Sichuan in China with the assistance of local veterinary practitioners. The samples were transported to the CADC-VDL at low temperatures. The 123 samples from apparently healthy pigs were collected during the epidemiological investigation of PRV, PRRSV, PPV, PCV2, and CSFV in China from 2010 to 2013. All 123 samples used in this study were identified as negative for the five pig pathogens listed above. A total of 48 samples from sick pigs with reproductive disease were collected in Beijing, and these included 6 mixed tissue samples from aborted fetuses, 4 serum or mixed tissues samples from piglets, 12 serum or mixed tissues samples from finishing pigs, and 26 serum samples from sows, which were collected in 2013 using procedures described previously [29].

### Nucleic acid extraction and routine detection

Total viral RNA and DNA were extracted directly from sera and tissue samples separately using a RNeasy Mini kit (Qiagen) and QIAamp viral DNA mini kit (Qiagen) following the manufacturer’s instructions. The RNA and DNA was re-dissolved in RNase- and DNase-free water and stored at −80°C until further processing. Conventional PCR or RT-PCR for PRV, PRRSV, PPV, PCV2, CSFV, swine influenza virus (SIV), Japanese encephalitis virus (JEV), and *Brucella suis* were carried out at the CADC.

### SISPA

Random detection of unknown virus genomic DNA or RNA from serum samples was performed using SISPA as previously described with some modifications [6]. To detect viral RNA, first-strand cDNA synthesis was performed using SuperScript™ III reverse transcriptase (Invitrogen, Carlsbad, CA, USA) primed with 5 pmol random hexamer primer 42°C for 1 h. The second-strand cDNA was then synthesized by adding 1 U of RNase H and 30 U of DNA polymerase I (Invitrogen) at 12°C for 1 h followed by 1 h at 22°C. To detect viral DNA, a complementary strand was generated by the incubation of extracted DNA with Klenow fragment (exo-; New England Biolabs, NEB, USA) and 5 pmol of random hexamers at 37°C for 1 h. Double-stranded DNA samples were then digested with the restriction enzyme TaqI (Takara, Dalian, China). Adaptors composed of hybridized oligonucleotides Taq24 (5′ ATCCCTCGGATAGCACAGTTGCCT3′) and NTaq11 (5′CGAGGCAACTG3′; 80 pmol) were then ligated to the restricted DNA using T4 DNA ligase (New England Biolabs, NEB) (1). Two microliters of the ligation reaction were used to prime PCRs containing 50 pmol of Taq24 and 2.5 units of Taq polymerase (Promega Madison, Wisconsin, USA). Cycling conditions were as follows: 40 cycles, which consisting of 94°C for 1 min and 72°C for 3 min [21,22].

The Amplified PCR products were then analyzed by agarose gel electrophoresis. The distinct DNA bands were gel-purified with an E.Z.N.A.TM Gel Extraction Kit (OMEGA, USA) and ligated with pGEM®-T Easy Vector System (Promega). The recombinant plasmids were transformed into Escherichia coli Trans1 T1 competent cells (Transgen, China). A total of 45 positive clones were sequenced in both directions using universal primers T7 and SP6 promoter-specific primers with an ABI Prism 3730 DNA sequencer (Applied Biosystems, USA) at Invitrogen (Beijing, China). The sequencing reads were trimmed, clustered, and subjected to BLAST homology searching with sequences deposited in GenBank (BLASTn and BLASTx; http://blast.ncbi.nlm.nih.gov.blast.cgi).

### PCR detection of PPV6

According to the sequence obtained from SISPA, two primers (PPV6F: 5′-GTCAAAGTGGGAACCCAATTG-3′ and PPV6R: 5′-CCTGGACAGCAAGAAGAAATG-3′) were designed to amplify a 371 nucleotides region within the ORF2. All samples were screened for PPV6 by PCR. The thermal cycling conditions were 94°C for 3 min, followed by 35 cycles of 94°C for 30 s, 52°C for 45 s, 72°C for 45 s, and a final elongation step at 72°C for 10 min. Finally, the PCR products were analyzed on 1.5% agarose gel electrophoresis ultraviolet imaging. Positive samples were determined with 371 bp amplified products.

### Whole genome sequencing

To dissect the phylogenetic position of PPV6, five representative viruses were selected for whole-genome sequencing. Four isolates (i.e., BJ2, SC, JS, and TJ) from healthy pig herds were selected to represent four geographical regions in China (i.e., Beijing, Sichuan, Tianjin, and Jiangsu). Also, the PPV6-BJ strain was selected to represent the sick pig herd with reproductive failure in Beijing. First, based on SISPA-generated fragment sequences, diverging primers were designed to obtain the intervening portions of the genome. Second, the terminal sequences were then acquired using the 5΄ and 3΄ RACE method (Invitrogen, Cat. No. 18373 and 18374) [8]. For 5′RACE, the PPV6 genome was first linearly amplified (60 cycles, with 1 cycle consisting of 94°C for 30 s, 50°C for 30 s, and 72°C for 2 min) using *Taq* polymerase and PPV6-specific primer p5RACE (5′-TGCGCTTATCTTCATTCAGAC-3′). Amplification products were then purified and a poly(C) tail was added to the 3′ end using deoxycytidine and terminal deoxynucleotidyl transferase. The 5′ region was then amplified with 5 units of *Taq* polymerase using an abridged anchor primer and *Taq* polymerase (5 U). For 3′RACE, gene specific primers p3RACE (5′-TATGGCCCATGTAAACGCATC-3′) were designed based on the available partial sequence and was used in combination with the Oligo-dT anchor primer. RACE-PCR reactions were performed using *Taq* DNA polymerase for 60 cycles (1 cycle consisting of 94°C for 45 s, 55°C for 45 s, and 72°C for 2 min). The products were analyzed by agarose gel electrophoresis and then sequenced according to standard protocols. Third, the acquired sequences were assembled into a complete genome with the aid of Vector NTI Site 9.0 software (Invitrogen). Finally, the five near full-length genome data from 5 positive samples (i.e., PPV6-BJ, BJ2, SC, JS, and TJ) were deposited in GenBank under accession Nos. KF999681-KF999685.

### Phylogenetic analysis

Sequences alignments were performed using the ClustalX (Ver.1.81) program. A neighbor-joining (NJ) tree was constructed using MEGA version 6 software (www.megasoftware.net). Reliability of the NJ tree was calculated using 1,000 bootstrap replicates. In addition to the PPV6 viruses, the complete sequences of various other parvoviruses were obtained from GenBank.

### Virus isolation

Virus isolation of PPV6 was attempted in PK15 (swine kidney), Vero (African green monkey kidney), and Marc 145 cells, as previously described with modifications [30]. Cells were seeded at 1.0 × 10^5^ cells ml^−1^ and, after 1 h incubation, they were inoculated with sample. Cells were grown in DMEM supplemented with penicillin (100 U/ml) and streptomycin (100 μg/ml), and were incubated at 37 °C in a humid environment containing 5 % CO_2_. If no CPE was observed at 7 days post-inoculation, the plates were frozen and thawed once and the supernatants were inoculated on new cells for a second passage. Inoculated cells at each passage were also tested by PCR, as described above. If CPE and PCR were negative after four passages, the virus isolation result was considered negative.
